# Holistic Determination of Optoelectronic Properties using High-Throughput Spectroscopy of Surface-Guided CsPbBr3 Nanowires

**DOI:** 10.1021/acsnano.2c01086

**Published:** 2022-05-18

**Authors:** Stephen A. Church, Hoyeon Choi, Nawal Al-Amairi, Ruqaiya Al-Abri, Ella Sanders, Eitan Oksenberg, Ernesto Joselevich, Patrick W. Parkinson

**Affiliations:** † Department of Physics and Astronomy and Photon Science Institute, The University of Manchester, Manchester M13 9PL, United Kingdom; ‡ Department of Materials and Interfaces, Weizmann Institute of Science, Herzl St 234, Rehovot 7610001, Israel; ¶ Center for Nanophotonics, AMOLF, Amsterdam 1009 DB, The Netherlands

**Keywords:** high-throughput, metal−halide perovskites, energy dynamics, photoluminescence, nanowires

## Abstract

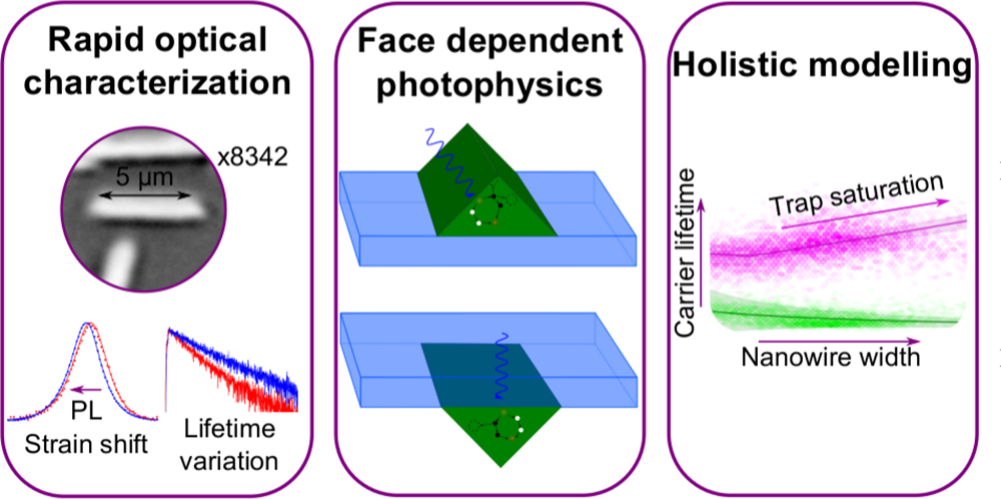

Optoelectronic micro- and nanostructures have a vast parameter space to explore for modification and optimization of their functional performance. This paper reports on a data-led approach using high-throughput single nanostructure spectroscopy to probe >8000 structures, allowing for holistic analysis of multiple material and optoelectronic parameters with statistical confidence. The methodology is applied to surface-guided CsPbBr_3_ nanowires, which have complex and interrelated geometric, structural, and electronic properties. Photoluminescence-based measurements, studying both the surface and embedded interfaces, exploits the natural inter nanowire geometric variation to show that increasing the nanowire width reduces the optical bandgap, increases the recombination rate in the nanowire bulk, and reduces the rate at the surface interface. A model of carrier recombination and diffusion ascribes these trends to carrier density and strain effects at the interfaces and self-consistently retrieves values for carrier mobility, trap densities, bandgap, diffusion length, and internal quantum efficiency. The model predicts parameter trends, such as the variation of internal quantum efficiency with width, which is confirmed by experimental verification. As this approach requires minimal *a priori* information, it is widely applicable to nano- and microscale materials.

Functional optoelectronic materials form the basis of a multitude of devices that are crucial to modern technology, including CCD detectors, photovoltaics, laser diodes, and LEDs. There is particular interest in optoelectronic micro- and nanostructures for increased density integration and enhanced performance with respect to planar devices. Some examples of these include micro-LED arrays for visible-light communication^1^ and nanolasers^2,3^ for on-chip photonic integrated circuits. In these structures, the functional performance is determined by many important optoelectronic properties,^4^ such as the diffusion length, bandgap, and defect density.^5^ The structural geometry, such as length, width, or shape, is also an important factor; this can be highly coupled to other parameters, making nanotechnology targets particularly challenging to develop.

Automation and high-throughput spectroscopy offer a means to harness the variation in properties across a large population of nano- or microstructures. This fundamental approach has been used for materials spanning tens of microns in length, down to micron scale.^4^ This allows correlations to be drawn between measured properties and can establish those with the greatest impact on performance.^6^ In this study, this approach is applied to holistically characterize microstructures by studying each individual element with multiple techniques, including photoluminescence spectroscopy, time-correlated single photon counting (TCSPC), and excitation power-dependent TCSPC, with a typical characterization time of a few seconds per NW. A self-consistent analysis is applied to this multimodal data set, of 15576 individual measurements, to correlate all of the measured properties and establish the coupling between geometry, strain, and carrier recombination processes. This allows the extraction of important parameters with statistical confidence, such as the bandgap, diffusion length, trap densities, and internal quantum efficiency (IQE).

The multimodal data-led approach is applied to surface-guided CsPbBr_3_ nanowires (NWs). This material has recently been demonstrated to have bright luminescence,^7^ strong waveguiding properties,^8^ and low lasing thresholds.^9^ These properties make the NWs ideal for applications in photodetectors,^10^ photovoltaics,^11^ and on-chip coherent light sources.^12,13^ Optical techniques have previously been applied to study spatially resolved degradation and recombination and strain effects in thin films of halide perovskites.^14,15^ However, the optical behavior of NWs demonstrates additional dependencies which increase the complexity of an experimental study. For example, the functional performance of these structures is strongly influenced by the strain^16^ and carrier trap densities, particularly at the surface; therefore fabrication processes are designed to control the material quality. However, these techniques do not control the NW geometry and often lead to variation in dimensions on the single NW level.^17^ The optoelectronic behavior of these NWs is therefore described by a complex, correlated, and multidimensional parameter space. The multimodal data-led approach is required to gain an understanding of the properties of this challenging material system by harnessing this inter-NW variation.

## Results and Discussion

### Experimental Results

**Figure 1:**
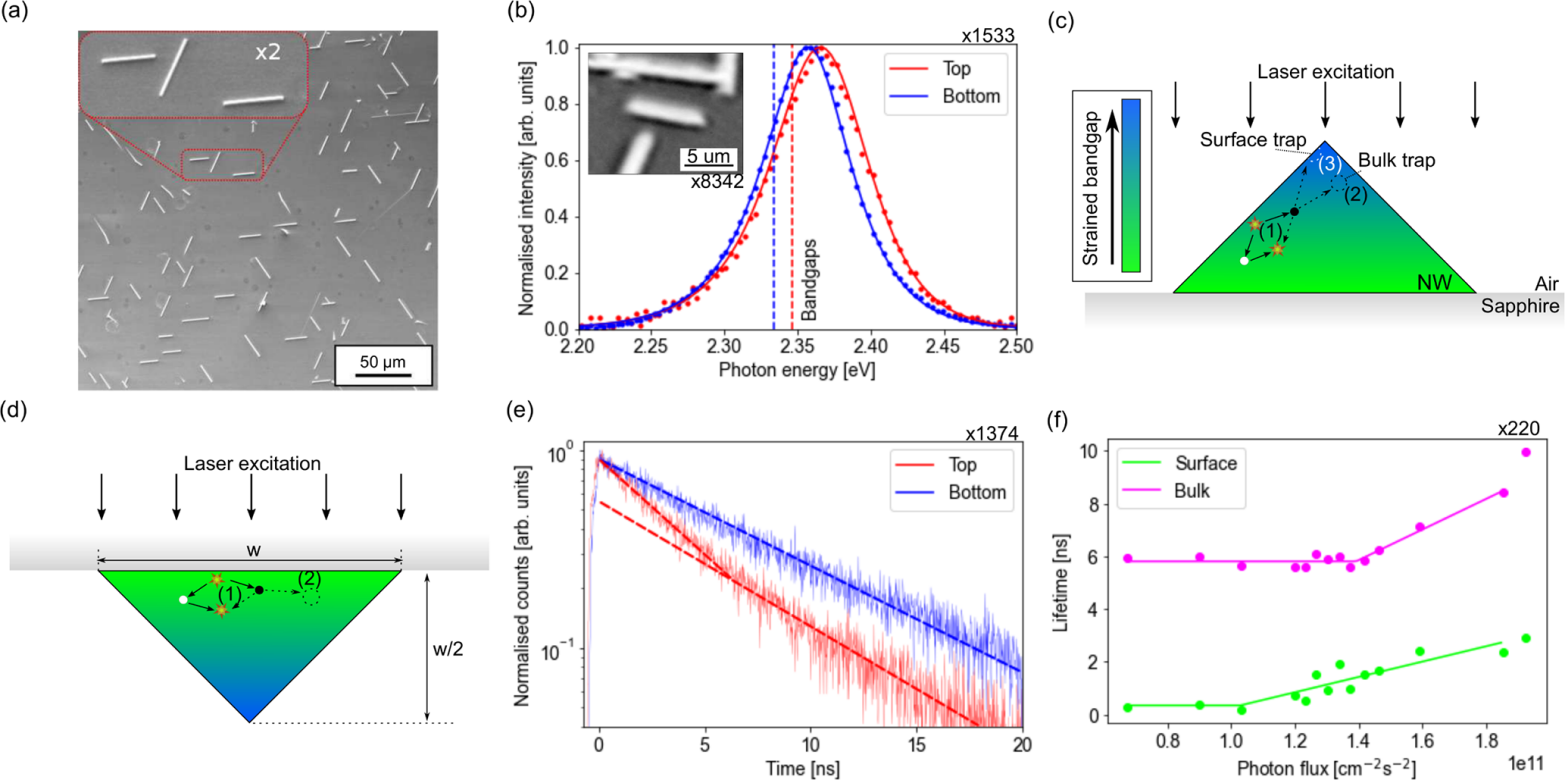
Single shot measurements of a NW subset. (a) SEM image of a small population of CsPbBr_3_ NWs showing the inter-NW variation of the length and width, along with the intra-NW uniformity of the width. Inset is a magnified image of 3 NWs. (b) PL spectra (dots) for top and bottom excitation of a single NW and the fit curve (solid lines) using a hybrid Urbach–Boltzmann given by eq S3. Optical microscopy image of the same NW (inset). (c) Schematic of the NW cross section when laser excitation occurs from the top interface. Carriers are generated at the air/NW interface and are either trapped at the surface or diffuse throughout the colored region before radiatively recombining or being trapped in the bulk. The bandgap varies throughout the cross section due to tensile strain at the NW/sapphire interface, resulting in a reduced bandgap at the base. (d) NW schematic when exciting occurs from the bottom, where carrier recombination is less sensitive to effects at the air/NW interface. (e) TCSPC data for top and bottom excitation of the same NW as (b). (f) Power dependence of the surface and bulk recombination lifetimes for a single NW. This data has been fit with a threshold model given by eq S5.

CsPbBr_3_ NWs were grown using a previously described recipe.^17^ Scanning electron microscopy (SEM) was performed on a small number of the NWs (252): a subset of these are shown in Figure 1a. The mean NW length, and standard deviation (SD), was (18 ± 10) μm, and the mean width was *w* = (1.2 ± 0.1) μm. NWs grown with this approach have an approximately cubic crystal structure: the substrate/NW interface is the (110) plane, and the NW/air interface consists of both the (010) and (100) planes.^17^ As a result, the NWs have an isosceles triangle cross section, i.e., with a height equal to half of the width. As discussed in ref (18), the cubic lattice is slightly distorted due to strain and lattice rotation effects. The NW width was hence obtained independently using atomic force microscopy (AFM) on 116 NWs, where the mean height, and SD, was measured to be *h* = *w*/2 = (0.6 ± 0.2) μm. More details are given in Figure S1.

Microscopy imaging and spectroscopy was performed separately on more than 8000 NWs, and an example of a single NW is shown in Figure 1b (inset). It takes approximately 0.1 s to locate a single NW and capture an image. PL spectra of this NW are also shown in Figure 1b: these spectra take approximately 1 s to acquire (including the time to move to the NW).

These spectra were fit with an emission model to extract the average PL bandgap (discussed in the Experimental Section). The bandgap differs by 13 meV when excitation occurs from the top and bottom, which are calculated to be (2.348 ± 0.001) eV and (2.335 ± 0.001) eV, respectively, provided with their standard errors (SE). This shift results from a lattice mismatch at the NW/substrate interface, leading to a reduction in the bandgap due to tensile strain at this interface.^17^ The tensile strain relaxes with distance from the interface;^18^ therefore, carriers recombining further from the NW/substrate interface emit higher energy photons: this is schematically illustrated in Figure 1c,d. When exciting from the top, the volume of material that the photocarriers can access spans a large range of strain environments due to the variation in proximity to the lower interface. Conversely, when excitation occurs from the bottom, the proximity to the lower interface is comparatively uniform and so the strain environments sampled are also uniform. This difference results in a larger degree of spectral inhomogeneity, which is shown in Figure S4a. These results demonstrate that the carrier diffusion length is smaller than the NW size, resulting in a nonuniform carrier distribution.

TCSPC decays taken from the same NW are shown in Figure 1e, illuminating from the top and bottom. Each TCSPC measurement can take up to 10 s to acquire. When excitation occurs from the bottom the decay is monoexponential with a lifetime, and SE, of (8.2 ± 0.4) ns. Excitation from the top results in a biexponential decay. In this experiment, the peak carrier density is kept low (around 1 × 10^16^ cm^–3^) to avoid bimolecular effects, and so the shape arises from two separate monoexponential processes with lifetimes, and SEs, of (3 ± 2) ns and (8 ± 2) ns.

The fast decay is only observed when excitation occurs from the top, and so it is associated with carrier dynamics at the air/wire surface.^19^ Similar biexponential results are observed from PL measurements on CsPbBr_3_ films,^20^ and since the slow decay is observed in both measurements it is tentatively ascribed to carrier dynamics in the wire bulk. These assignments are supported by carrier diffusion models, shown in Figure S2, which demonstrate that the majority of carriers recombine in the bulk volume of the NW. These results also suggest that the bottom interface may be passivated.

It has previously been established that nonradiative carrier traps can dominate the carrier recombination in CsPbBr_3_ materials.^21^ The carrier densities used in this study are in the regime where these traps may be saturating;^22^ therefore, this saturation process can be studied by varying the excitation power. An example of these power-dependent results from a single NW is shown in Figure 1f. Both lifetimes are constant at low excitation powers and increase above a threshold due to saturation effects. This observation is compatible with previous studies which utilize higher carrier densities and, therefore, observe longer carrier lifetimes than those reported here.^7,23^ The threshold and the rate of change of the lifetime differ for the bulk and the surface, which likely reflects differences in the carrier traps in these regions.

Top/bottom single-element measurements provide unambiguous insight into surface effects but provide no statistical strength or insight into correlation between parameters. Therefore, the full data set can be used to exploit the variation in width to extract the link between NW geometry and dynamics. The NW widths were measured using optical microscopy and calibrated using SEM: this resulted in the range of widths of NWs studied to be between 1.0 and 1.4 μm (more statistics are provided in Figure S1).

A total of 8342 NWs were imaged, taking a duration of 14 min to image the entire NW population. A subset of these NWs was studied for each measurement. The PL spectra were determined when exciting came from the top and bottom for a subset of 1533 NWs, taking 26 min in total for each experiment. The PL lifetimes were measured for a subset of 3744 NWs from the top and 1737 from the bottom, taking approximately 5 and 4 h, respectively. In total, it took approximately 11 h to record the 15576 individual measurements, with the vast majority of this time taken up by recording the TCSPC decays.

**Figure 2:**
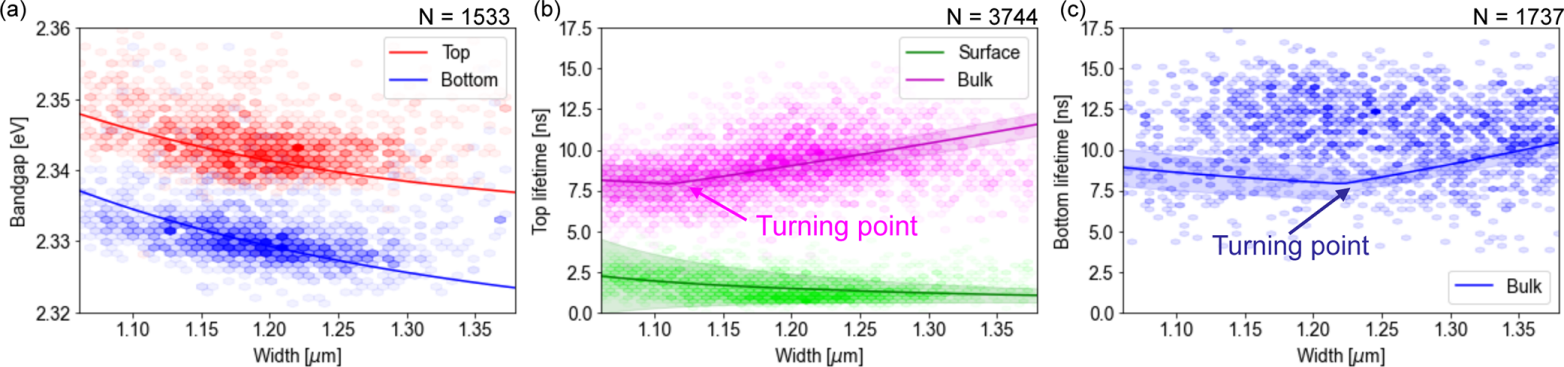
High-throughput optical results from the NW population (the shaded regions represent the SE on the model fit and *N* is the number of data points in each data set). (a) Bandgap obtained from fitting the PL spectra from 1533 NWs, correlated with the NW width. Data is shown when exciting occurs from the top and bottom. The solid lines are fits of equations in the Experimental Section to the data. (b) Lifetimes, measured with excitation from the top, for the surface (fast) and the bulk (slow) recombination correlated with the width of 3744 NWs. The lines are fits of equations in theExperimental Section to the data. The wide transparent area represents the 1σ uncertainty of the model fit. (c) Bulk lifetimes, measured from the bottom as a function of the width of 1737 NWs. Turning points in the rates and carrier densities occur due to the onset of carrier trap saturation. The errors on the lifetime data have median values of 0.3, 0.5, and 0.04 ns for the top bulk and surface and bottom, respectively: these numbers give reduced χ^2^ values of 1.5, 0.1, and 5.8.

The correlation between PL-determined bandgap and NW width is shown in Figure 2a: in all cases the bottom illumination is red-shifted relative to the top illumination. This energy shift depends on the tensile strain at the bottom interface, and the recombination positions of the carriers. As the NW width is reduced, the bandgap blueshifts in both measurements. This has been previously attributed to lattice rotation effects, which increase the bandgap due to changes in the lattice bond angles.^18^ In a single NW, the lattice rotation is uniform, and so the overall effect is that the tensile strain relaxes and the bandgap increases with distance from the NW base, as shown in Figure 1e. The rotations increase in magnitude for narrower NWs,^17^ resulting in larger bandgaps and energy shifts of up to 60 meV for NWs of 1 μm width. There is a good correlation between our geometrical measurements and a previously determined relationship between geometry and emission energy.^17^

The lifetimes for top and bottom illumination are shown in Figure 2b,c, respectively. The excitation-power dependence of the dynamics for a subset of 220 randomly chosen wires was also studied, and is presented in Figure S4 in the S.I. For the vast majority of NWs, bulk and surface recombination lifetimes are observed when excitation occurs from the top, and only the bulk lifetime is observed from the bottom. As the NW width increases, the bulk lifetime becomes longer and the surface lifetime becomes shorter. This relationship between lifetime and NW width was investigated further with a model for carrier recombination.

### Model Results

To understand the impact of NW width on carrier dynamics, a self-consistent model of diffusion and recombination was considered to explain the ensemble. Due to a small optical absorption length at the excitation wavelength (approximately 100 nm^24^), carriers are generated close to the air/NW surface or the NW/substrate interface for top and bottom illumination, respectively. These carriers can then diffuse throughout the NW and may recombine radiatively: alternatively, the carriers may be trapped and recombine nonradiatively at the top interface or in the bulk.^23,25^ Transmission electron microscopy measurements do not observe the formation of any strain-related defects at the bottom interface,^18^ and the TCSPC measurements also show no evidence of an additional recombination mechanism. For this reason, the bottom interface is treated like bulk material in the model. The model makes no assumptions regarding the nature of the carriers that dictate this behavior, and also no assumptions about the nature of the carrier traps. Candidates for these traps include dangling bonds at the surface,^26^ Pb vacancies, and Br interstitials.^21^

The model relies on the observation that carrier diffusion occurs on a time scale faster than recombination.^27^ A schematic of this process is shown in Figure 1c,d. Carrier diffusion is accounted for using a time-dependent Monte Carlo model that is shown in Figure S2. This produces a distribution of carrier recombination locations in the NW that is used to calculate the carrier densities and dynamics, as described in the Experimental Section. Table 1 provides a summary of the most important extracted optoelectronic parameters from this model: all uncertainties quoted in this section are the SE extracted from the model.

**Table 1:** Optoelectronic Parameters Derived from the Model, With Their SEs, Compared with Literature Values from Single-Shot Studies (Where Available)^c^

parameter	unit	value	lit. value
bulk trap density: *N*_V_	10^16^ cm^–3^	8.6 ± 0.4	<15^a^,^28^
surface trap density: *N*_S_	10^16^ cm^–3^	7.1 ± 0.3	<12^29^
unstrained bandgap: *E*_g_	eV	2.4 ± 0.1	2.36^30^
diffusion length: *L*_D_	μm	0.25 ± 0.02	9.2^b^,^31^
IQE (top)	%	0.7 ± 0.1	
carrier mobility: μ	cm^2^ V^–1^ s^–1^	0.8 ± 0.1	35^c^,^7^

a Reports for spin-coated CsPbBr_3_ LEDs.

b Measured for CsPbBr_3_ single nano-crystals.

c Reports for carrier diffusion along the long axis of CsPbBr_3_ NWs.

The PL bandgap varies with NW width and carrier recombination location in the wire. The model allows us to separate the effects of tensile strain and lattice rotations from the data in Figure 2a, using the equations defined in the Experimental Section. As the NW width increases, the lattice rotation effects reduce and the bandgap redshifts (equally for top and bottom excitation). This lattice rotation effect accounts for a bandgap shift of 12 meV across the NW ensemble, which is comparable to values previously measured using photoluminescence on a small number of NWs of this size.^17^ Increasing the width also increases the average separation between carriers when exciting occurs from the top and bottom; this causes a larger shift between the two bandgap measurements. This is a comparatively small effect, accounting for an additional 2 meV bandgap shift across the ensemble. The multimodal fit determines the unstrained bandgap to be (2.4 ± 0.1) eV, which is consistent with literature values.^30^ It is notable that this result cannot be obtained from a single-wire measurement because none of the NWs in this study have unstrained emission.

The calculated ambipolar diffusion length is (0.25 ± 0.02) μm, which is smaller than best-in-class literature values.^31,32^ This is smaller than the NW cross section, validating the assumption that diffusion will be an important in carrier recombination behavior, and that the carrier distribution is nonuniform. Additionally, the carrier mobility is (0.8 ± 0.1) cm^2^ V^–1^ s^–1^, comparable with catalyzed vapor–liquid–solid grown NWs.^33^ However, there is a wide spread of mobilities reported in the literature due to variation in sample quality and experimental approaches.^31,34^ Our mobility is smaller than previous reports on NWs grown using the same method, studying diffusion along the NWs.^7^ This discrepancy may partially be due to differences in excitation conditions, and carrier densities, as well as the nature of the high-throughput study, which accounts for the entire NW population, including those of poorer quality. There is also the possibility of mobility anisotropy along and across the NW cross section, due to strain or compositional variation in each direction.^15^

The model fits to the lifetime measurements are shown in Figure 2b,c. The model accounts for the trends in lifetime with NW width when exciting occurs from the top. The larger relative uncertainties on the fast lifetimes are reflective of the increased uncertainties on the lifetimes measured from each NW. The bottom lifetime data has a greater spread than is accounted for in the model; this may be experimentally driven, arising from challenges in focusing the microscope objective through the sapphire substrate. This data still provides a useful constraint on the holistic model.

The observed lifetime trends are primarily influenced by trap saturation effects, which allow the density of traps to be calculated. At the surface this is (7.1 ± 0.3) × 10^16^ cm^–3^, which comparable to the trap density in the bulk, (8.6 ± 0.4) × 10^16^ cm^–3^. However, the surface traps lie within a small depth, leading to a high areal density of (4.3 ± 0.7) × 10^10^ cm^–2^ and increasing the surface recombination rates. Accounting for the traps in the surface and bulk regions, the average number of traps in a single NW is (6.3 ± 0.3) × 10^8^. For comparison, an excitation pulse with 2 × 10^11^ photons.cm^–2^ generates (2.8 ± 0.1) × 10^8^ carriers in a NW, averaged over the NW widths considered in this study. The excitation conditions in this experiment are therefore sufficient to study trap saturation effects and the trap densities lie in the range of literature values.^28,29^

The holistic approach, which analyzes and correlates all the measured properties for each single element in the ensemble, has therefore determined material parameters that are compatible with literature values obtained from single-shot measurements (where available), and has established values with statistical confidence that represent the entire NW population.

**Figure 3:**
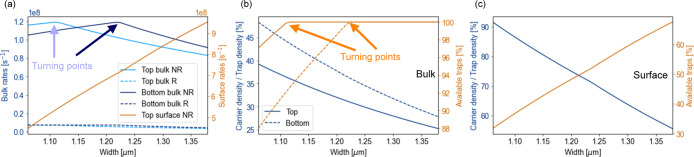
Derived parameters from the global model fit for NWs with different widths: (a) calculated radiative (R) and nonradiative (NR) rates. Turning points in the rates and carrier densities occur due to the onset of carrier trap saturation. (b) Carrier and unoccupied trap densities in the bulk when exciting occurs from the top and bottom, normalized to the trap density. (c) Carrier and unoccupied trap densities at the surface when exciting occurs from the top, normalized to the trap density.

The model can describe the variation in the carrier recombination rates for different NW widths, as shown in Figure 3a. The mean nonradiative rate at the surface is (7 ± 1) × 10^8^ s^–1^ and dominates over the other processes. This is compatible with conclusions from other studies.^25^ The mean bulk nonradiative rate is (1.0 ± 0.1) × 10^8^ s^–1^, which is much faster than the mean radiative rate of (5 ± 1) × 10^6^ s^–1^. Therefore, nonradiative effects dominate the carrier dynamics, as predicted by previous studies.^23^

The lifetime variation with NW width can be explained by considering the effect of trap and carrier densities. As shown in Figure 3b,c, as the wire width increases the carrier density reduces at the surface and in the bulk. As the excitation conditions are constant for each measurement, this is due to an increased wire volume and surface. This causes a reduction in the radiative rate with increasing wire width in Figure 3a.

The nonradiative rate is influenced by the carrier density and the effective trap density, given by the trap density minus the saturated trap density. As the carrier density increases above a threshold, the effective trap density will reduce due to saturation. This can be seen for the narrowest NWs in Figure 3b. The overall change in the nonradiative rate will be a combination of these effects.

Figure 3a shows that the bulk nonradiative rate from the top increases with width up to 1.12 μm. This occurs because the trap density is limiting the nonradiative rate, and there is an increase in the available traps. The trap saturation can be as high as 13% in these NWs. For wider NWs, there is effectively no trap saturation, and so the available traps remains constant at 100%. The nonradiative rate is now limited by carrier density and so drops with increasing width. This results in the turning points in the model, and the lifetime increase observed in Figure 2b. As the turning point is related to trap saturation, it is also dependent upon the excitation fluence used in the experiment: for example, a higher fluence would cause a greater degree of trap saturation and shift the turning point to wider NWs. At the surface, the carrier density is sufficient to saturate a portion of the surface traps for all NW widths, as shown in Figure 3c, leaving between 35% and 70% of traps available for capture. As a result, the nonradiative rate increases with wire width, and the lifetime in Figure 2b drops.

When excitement occurs from the bottom, the carrier density in the bulk is higher due to stronger coupling of the incoming light, as demonstrated in Figure S1. The higher carrier densities mean that the radiative rate is, on average, 25% faster at the bottom. However, this change is insignificant when compared to the nonradiative rates. Additionally, more bulk traps are saturated for the same NW width. This shifts the turning point in the nonradiative rate to wider NWs. The higher carrier density partially balances out with the trap saturation, and so the bulk nonradiative rate from the bottom is comparable to that from the top, demonstrated in Figure 3a. As shown in Figure 2c, this means that the fit to the bottom lifetime is of a similar shape.

The model can also be used to predict optoelectronic behavior of the NWs with statistical confidence. Equation 8 in the Experimental Section was used to calculate the average recombination IQE when excitement occurred from the top, which was found to be (0.7 ± 0.1)%. This result was independently verified using temperature-dependent PL to estimate the IQE of a small number of NWs. The mean room temperature IQE was measured to be (1.1 ± 0.3)%, which is consistent with the model calculation. Since the NWs have a significant variation in geometric parameters and have a strongly nonuniform coverage across the substrate, these individual single-NW IQE measurements are more meaningful that an ensemble PL-quantum yield measurement. More details of the temperature-dependent PL, along with the variation of the IQE with width are shown in Figure S4.

The IQE can be increased by exciting the carriers at the bottom of the wire, which will reduce the number of carriers that diffuse to the surface. This will reduce the overall rate of nonradiative recombination: the model predicts that the IQE will increase to (5.8 ± 0.4)%. This prediction was again confirmed using temperature dependent PL, which is shown in Figure S6 to be (7 ± 1)%.

## Conclusion

This paper reports on a multimodal high-throughput spectroscopy technique that harnesses the variation in geometry among a nanomaterial population to extract optoelectronic properties and performance. This is achieved through measurements of optical images, PL spectra and PL decays of the same nanomaterials when illuminating from two different directions. The analysis of global trends across the population enables the extraction of a multitude of important optoelectronic parameters with statistical confidence that are comparable to separately determined values in the literature.

The technique has been applied to CsPbBr_3_ NWs, only requiring an independent measurement of the cross-section shape and the refractive index. The PL bandgap increases with reduced NW thickness due to increasing bond rotation effects and reduces at the wire/substrate interface due to tensile strain. Nonradiative recombination at defects at the air/NW surface is the dominant carrier recombination process, while nonradiative recombination at bulk defects and radiative recombination are also observed. As the NW width increases, the bulk recombination lifetime increases and the surface recombination lifetime decreases. A self-consistent model of carrier diffusion and recombination was developed to explain these trends and showed that they are due to changes in carrier density, and trap saturation, with NW width. Furthermore, the model extracts the carrier diffusion length, mobility, trap densities and bandgap from the multimodal data, giving results which are compatible with the literature. Crucially, the technique is able to accurately predict the IQE of carrier recombination, and how this changes when illuminating from different directions. As minimal *a priori* information is required, this approach provides a data-driven methodology to explore nanomaterial systems.

## Experimental Section

### Sample Growth

The surface-guided NWs were synthesized via a vapor-phase method on *c*-plane sapphire substrates. A spectroscopic hot plate (Linkam THMS600) is used, with a round stage size of ∼2 cm radius, over which a silicon wafer is placed as a holder for chunks of molten CsBr and PbBr_2_ powders (both 99.999%, purchased from Sigma-Aldrich). The sample was placed face down over an aluminum ring spacer, such that the distance from the chunks is ∼500 μm. The stage is then heated to 420 °C with a rate of 30 °C  min^–1^ and kept at the NW growth temperature for 5–10 min, after which the stage is cooled down to room temperature at a rate of ≈100 °C  min^–1^.

### Scanning Electron Microscopy

SEM was performed using a Quanta250 FEG microscope to acquire images of the NWs. The images were obtained using a secondary electron detector at 11.3 mm working distance and 5 keV acceleration voltage. Images acquired at 500× magnification show ∼120 NWs per image.

### High-Throughput Spectroscopy

NWs on their substrates were placed into a home-built microscope with a quasi-confocal setup for optical excitation and light collection. A motorized *xyz* translation system was used for brightfield microscopy with an optical resolution of approximately 1 μm. A machine vision camera was used to identify NWs by their geometrical properties (width, length, orientation, etc.). A total of 8342 NWs were identified using this method, taking approximately 0.1 s per NW. The NW widths in this study were close to the resolution of the microscope, and so these measurements were calibrated using SEM and AFM, as shown in Figure S1.

Room-temperature photoluminescence spectroscopy was performed using a 405 nm wavelength pulsed laser, with a 50 ns pulse period and 80 ps pulse duration, to excite the NWs in their center. The laser spot was focused to an elliptical spot size of 8 μm by 21 μm and the photon flux at the NW was estimated to be 2 × 10^12^ /cm2/pulse. A long-pass filter was used to remove the excitation light and the luminescence was focused onto a fiber connected to an Ocean Optics QE65000 spectrometer with a spectral resolution of 0.3 nm. This experiment was automated to sequentially measure the PL of the full NW population, taking approximately 1 s per NW, including movement time. A schematic of this system is shown in Figure S2.

The PL time decays of NWs were measured by routing the output optical fiber to single photon avalanche diodes connected to a Picoharp HydraHarp400 TCSPC system. The combined experimental setup has a time resolution of 200 ps, and it takes between 1 and 10 s to measure a single wire, including movement time. The excitation power was varied using a motorized ND filter wheel. Power-dependent TCSPC was measured for a subset of 220 NWs.

PL measurements were obtained by exciting NWs from the top and from the bottom. This was achieved by flipping the sample substrates in the microscope and repeating the machine vision imaging to identify another set of NWs, and repeating the high throughput spectroscopy. The NWs that were present in both populations were identified using an approach similar to reference.^35^ This resulted in 1533 NWs where both sets of measurements were performed.

### PL Fitting

The PL spectra were fit with a model assuming that photogenerated carriers rapidly cool to the conduction and valence band edges and occupy a thermal distribution in a 3D density of states. This density of states was modified to include an Urbach tail at low energies and was convoluted with a Gaussian to account for inhomogeneity in the NW and the system resolution. More details of the fitting can be found in Figure S3.

### TCSPC Fitting

All PL decays were fit with both a monoexponential and a biexponential fit. The reduced chi-squared of these fits was assessed to determine if the measurements contained one or two decays. The same procedure was applied to the power dependence of the decays to obtain how the lifetimes vary with excitation power. A linear threshold model was fit to the lifetime vs excitation power data to extract the threshold power and the rate of lifetime change above the threshold, to constrain the recombination model. More details and examples of the TCSPC fitting can be found in Figure S3.

### Recombination Model

A quasi-steady-state model for photon absorption, carrier diffusion, and recombination was developed to fit the PL and TCSPC data. This includes the PL bandgap data and the PL lifetimes from Figure 2. The model is also constrained by the power dependence of the PL lifetimes and the integral of the PL decays, which are shown Figure S4. The model only requires prior knowledge of the cross-sectional shape and the refractive index of the material.

This model accounts for geometrical factors in the NW cross section, which differ when they are excited from the top or the bottom, as shown in Figure 1. COMSOL modeling is used to determine the average fraction of incident light which is absorbed in the NW, as shown in Figure S1, ⟨abs⟩. The number of carriers generated in the NW, *N*_0_, is given by eq 1
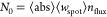
1where ⟨*w*_spot_⟩ is the average width of the excitation spot, *n*_flux_ is the photon flux of the excitation spot and *N*_0_ is in units of cm^–1^, which is low enough to ignore Auger recombination. As the absorption length is ∼100 nm,^24^ the calculations assume that these carriers are generated at the surface.

Carrier diffusion occurs on sub ns time scales:^27^ the model therefore assumes that carriers diffuse throughout the NW cross section before recombining, with a characteristic diffusion length *L*_D_. This diffusion is modeled using a Monte Carlo simulation of nearest neighbor hopping, the details of which are shown in Figure S2. Carriers that stop diffusing within a certain distance from either interface, *L*_surface_, are trapped at that interface: this is used to calculate an occupation factor *B*, which represents the proportion of carriers at the top surface, the bottom interface and in the bulk volume. The volume (*V*, cm^2^) of each of these regions is geometrically calculated and used in eq 2 to find the photogenerated carrier densities (*n*, cm^–3^):
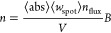
2Equations 1 and 2 apply to illumination from the top and bottom, with different values of ⟨abs⟩ and *B*. At the top surface, nonradiative recombination dominates;^23,25^ it is therefore assumed that all of the carriers at the interfaces are trapped. In the bulk volume, carriers are either free or trapped. The carrier densities are calculated using eq 3 and 4:
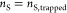
3
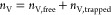
4The free carriers undergo radiative recombination with a rate proportional to the density of free carriers squared, and is assumed to be negligible at the top surface. Nonradiative recombination is mediated by the effective trapping rate, which is proportional to the density of unoccupied traps, *n*_t, eff_ and the density of free carriers. The total recombination rates are given by eqs 5 and 6
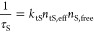
5
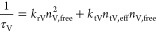
6where *k* are the rate constants and τ is the recombination time measured by TCSPC. Power-dependent TCSPC shows how these lifetimes increase when the excitation power increases above a threshold. This allows the model to constrain the trap density in each region using eqs S6–S10.

The bandgap of the NW is red-shifted due to tensile strain and is blueshifted by lattice rotation effects.^18^ These rotation effects are uniform throughout an individual NW and are stronger in narrower NWs.^18^ The tensile strain relaxes with distance from the NW base such that it is unstrained at the top. Therefore, carriers generated by top and bottom illumination will experience the same lattice rotation effects, but different degrees of strain, as illustrated in Figure 1c.

The average bandgap of carriers is modeled using the Monte Carlo diffusion model. The position of all the carriers in the ensemble is used to find the average distance from the bottom interface, *y*_av_. This distance is then included in an empirical equation that is fit to the data in Figure 2a. A simplification is made to the case in reference,^18^ assuming that the tensile strain relaxes linearly with distance from the bottom interface and is unstrained at the top apex. An exponential variation of energy with NW width is assumed, with constant *L*_rot_, due to lattice rotation effects. The bandgap is given by eq 7
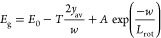
7where *E*_0_ is the unstrained bandgap of the NW, *A* is a scaling factor, *w* is the NW width, and *T* is the energy shift due to tensile strain at the base.

This model produces 13 fitting parameters in total. A detailed description of these parameters is provided in the SI. The fitting parameters can be used to derive values for the IQE, defined as the total radiative recombination rate, divided by the total decay rate, this is given by eq 8:
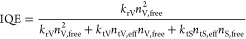
8Note that, for illumination from the bottom, *n*_S,free_ = 0. The carrier mobility, μ, can also be calculated using eq 9:
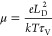
9

### Additional Techniques

To supplement the high throughput measurements, a subset of NWs were studied using AFM. Temperature dependent PL was also used to estimate the IQE. The coupling of the excitation beam into the NWs was studied theoretically and carrier diffusion was simulated using Monte Carlo methods. Details of these techniques are provided in the SI.

## Supporting Information Available

AFM of a subset of the NWs compared with SEM and optical microscopy, temperature dependent PL to estimate the IQE and additional statistics from the NW population. Details of the models for PL and TCSPC fitting, theoretical analysis of light coupling into the NWs, Monte Carlo simulations of carrier diffusion and a summary of the parameters in the holistic model (PDF)

## Notes

**CRediT author statement.****Stephen Church**: Formal analysis, Investigation, Methodology, Software, Visualization, Writing - original draft. **Hoyeon Choi**: Investigation. **Nawal Al-Amairi**: Investigation, Writing - review and editing. **Ruqaiya Al-Abri**: Investigation, Writing - review and editing. **Ella Sanders**: Resources, Writing - review and editing. **Eitan Oksenberg**: Writing - review and editing **Ernesto Joselevich**: Funding acquisition, Resources, Writing - review and editing. **Patrick Parkinson**: Conceptualization, Data curation, Funding acquisition, Methodology, Software, Supervision, Writing - review and editing.
