# Supplementary material for: Holistic Determination of Optoelectronic Properties using High-Throughput Spectroscopy of Surface-Guided CsPbBr3 Nanowires
Source: ACS Nano. 2022 May 18;16(6):9086–94. doi: 10.1021/acsnano.2c01086 (PMC9245348; doi:10.1021/acsnano.2c01086)
Supplement: Supplementary file 1 — nn2c01086_si_001.pdf [file nn2c01086_si_001.pdf]

Supporting Information for Holistic Determination of Optoelectronic Properties using High-Throughput Spectroscopy of Surface-Guided CsPbBr3 Nanowires

Stephen A. Church,*,† Hoyeon Choi,† Nawal Al-Amairi,† Ruqaiya Al-Abri,† Ella Sanders,‡ Eitan Oksenberg,¶ Ernesto Joselevich,‡ and Patrick W. Parkinson*,†

†*Department of Physics and Astronomy and Photon Science Institute, the University of Manchester, Manchester M13 9PL, United Kingdom*

‡*Department of Materials and Interfaces, Weizmann Institute of Science, Herzl St 234, Rehovot 7610001, Israel*

¶*Center for Nanophotonics, AMOLF, Amsterdam 1009 DB, the Netherlands*

E-mail: stephen.church@manchester.ac.uk; patrick.parkinson@manchester.ac.uk

Scanning electron microscopy and atomic force microscopy

Scanning electron microscopy (SEM), optical microscopy and atomic force microscopy (AFM) were used to measure the lengths and widths of nanowires (NWs). Example images for these techniques are shown in Figure S1a-c. The SEM and optical results are obtained using the systems described in the main paper. AFM was performed using a Nanosurf system in tapping mode with a Tap190Al-G probe. The maximum heights of the NWs were measured and the width was calculated by dividing the values by two, due to the triangular cross section.[1] Histograms of these results are shown in Figure S1d,e.

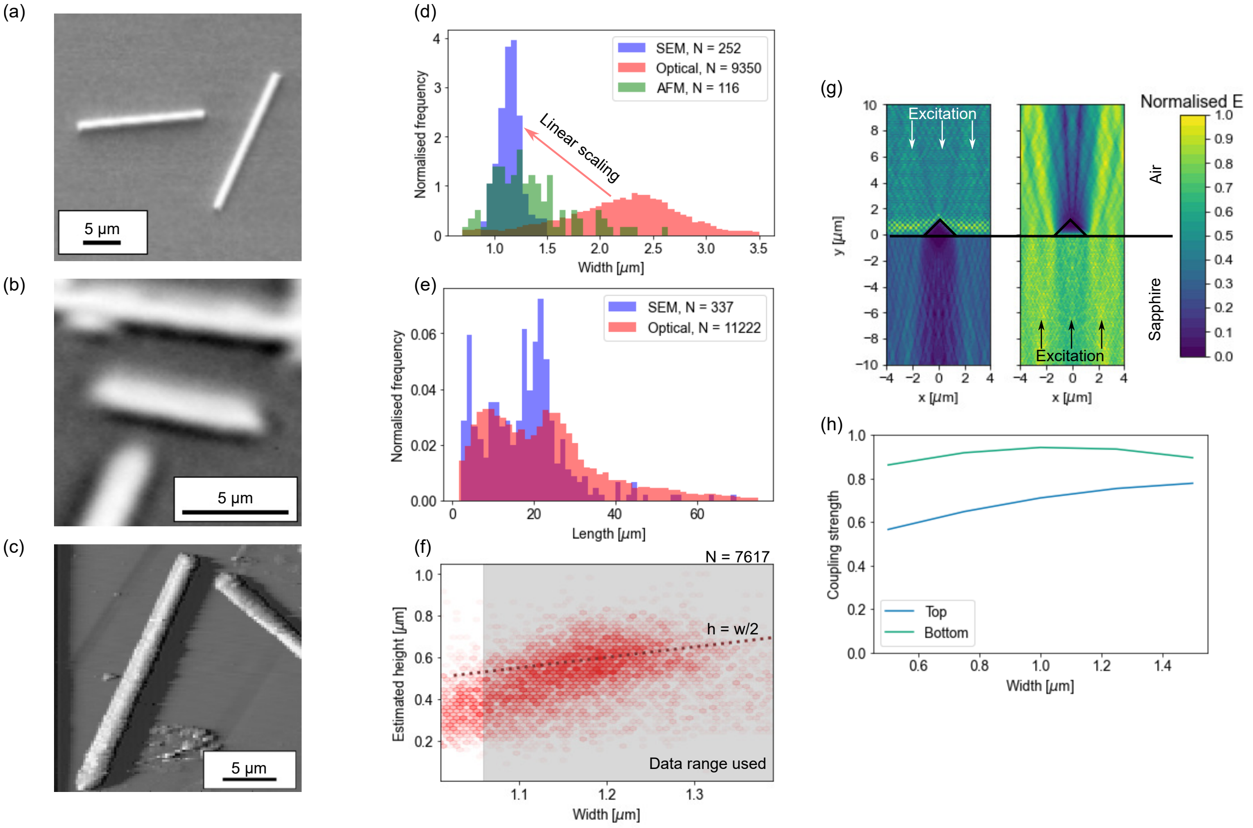

Figure S1: A summary of the geometrical characterisation and light coupling modelling of NWs. Images of $CsPbBr_3$ NWs taken using (a) SEM, (b) optical microscopy and (c) AFM. (d) A comparison of the width measurements for NWs using different techniques, 252 NWs were studied with SEM, 9350 using optical microscopy and 116 using AFM. (e) A comparison of the NW lengths using SEM and optical microscopy, 337 NWs were studied with SEM and 11222 using optical microscopy. (f) The correlation between the height, estimated from the PL peak energy, and the width for 7617 NWs. The shaded region represents the range of NWs considered in this study. The dotted line is a guide to the eye for h = w/2. (g) The normalised electric field distribution when exciting a NW from the top and bottom. (h) The coupling strength of light into NWs with different widths when exciting from the top and bottom.

The width distribution is comparable for SEM and AFM results, with median, and standard deviation (SD), values of (1.2 ± 0.3) µm and (1.2 ± 0.4) µm respectively. This provides evidence for the triangular cross sectional shape previously reported.[1] The NW widths for optical measurements are significantly larger, with median and SD values of (2.2 ± 0.6) µm. These larger values are because the widths are of the same order as the resolution of the optical microscope. To account for this a linear scaling was applied to the

optical widths in order to replicate the SEM histogram.

The NW length distribution is similar for SEM and optical results: the median and SD of the optical length was (21 ± 1) µm and this was (18 ± 1) µm in the SEM results. The distribution is bi-modal, with peaks at 9 µm and 24 µm in the optical measurements. The heights of the NWs were also estimated using a previously determined empirical relationship between emission energy when illuminating from the top and nanowire height.[2] A polynomial was fit to this previously reported data and applied to the PL spectra of 7617 NWs. The results of this conversion are shown in Figure S1f. As the NW width approaches the diffraction limit, the linear-scaling approximation breaks down due to an increase in the relative uncertainty in the width. We determined a validity range for our linear-scaling by performing a series of linear fits to the height-width relationship for different ranges of NW widths. We found that the h = w/2 relationship holds for NWs wider than 1.06 µm, and thus we selected these wires for further experimentation. These NWs represent 90 % of the total population demonstrate the consistency between results from previous studies on the same wires ,[2] the AFM widths (calculated from the height measurements) and the width calibration procedure using SEM and optical microscopy.

For narrow NWs, the estimated height, and SE, is (0.15 ± 0.10) µm less than that expected from the $h = w/2$ relation. This may be because these wires lie close to the resolution limit of the optical microscope: the width of the NWs in this region may therefore be over-estimated and these wires were removed from any analysis.

Light coupling modelling

The coupling of light into the NWs was calculated using the quadratic finite-element-method for different NW widths. A triangular cross section of an ideal NW was modelled in two dimensions with widths ranging from 0.5 µm to 1.5 µm. The NW was sandwiched between a sapphire substrate and the air. To account for the elliptical excitation spot, these calculations were repeated for modelling spaces with widths of 8 µm and 21 µm, each representative of

an axis of the ellipse. The calculations were also repeated for top and bottom illumination. Example modelling geometries are shown in Figure S1g. The real and imaginary refractive indices of the NW were defined as 2 and 0.32 respectively[3,4] and the refractive index for the sapphire was 1.77.[5] COMSOL multi-physics was used to perform the calculations, using the "electromagnetic wave, frequency domain (ewfd)" package to solve Maxwell's equation in three dimensions, given by Equation S1:

$$\nabla \times (\mu_{\mathrm{r}}^{-1} \nabla \times \mathbf{E}) - \omega^2/c_{\mathrm{o}}^2 (\epsilon_{\mathrm{r}} - \frac{j\sigma}{\omega \epsilon_{\mathrm{o}}}) \mathbf{E} = 0, \tag{S1}$$

where $\boldsymbol{E}$ is the electric field, σ is the electrical conductivity, ω is the angular frequency, μ_r and ϵ_r are the relative permeability and permittivity respectively. The results were averaged for electric field polarisation in and out of the plane.

The simulation accounts for reflection and transmission of light at each interface, scattering and diffusion of light from the interfaces, and absorption of light in the NWs. An example calculated electric field distribution is shown in Figure S1g. The proportion of the incident light intensity which is absorbed by the NW was calculated by considering the difference in intensity of the excitation and after the interaction. The intersection between a single NW and the excitation spot will vary depending on the NW orientation: to approximate this effect the result was averaged between the 8 µm and 21 µm configurations. To calculate the light coupling strength, the absorption was divided by the absorption of a block of perfectly absorbing material with the same dimensions as the NWs. The coupling strengths are shown in Figure S1h. The bottom coupling also includes the reflectivity of the initial air/sapphire interface at the bottom of the sample, calculated to be 0.077.

The coupling strength when illuminating from the bottom remains approximately unchanged with wire width, with a value and SD of (0.91 ± 0.03) The coupling strength is high due to smaller changes in refractive index at the two interfaces, reducing the Fresnel reflection at the interfaces. The coupling is constant because light is normal to a flat interface, which

is unchanged by changing the width. However, for top illumination the coupling strength is 0.78 for wire widths close to 1.5 μm and reduces for smaller widths. The reduced coupling is partially due to enhanced reflection from a larger change in refractive index. Additionally, scattering from the apex of the triangle increases for smaller NWs.

Carrier diffusion model

The measured properties of the NWs depends on where in the NW the photogenerated carriers recombine. This recombination location affects the recombination energy, and thus the measured bandgap, and impacts the recombination mechanism for the carriers (i.e. non-radiative at traps or radiative). The distribution of recombination locations is determined by the NW geometry and size, the carrier diffusion length and the effective thickness of the surface layers. This is a complex 2-dimensional diffusion problem, with no trivial analytical solution.

The recombination distribution is calculated for a NW using a Monte-Carlo approach. In this method, 40,000 carriers are injected individually at random points on the top surface of a NW cross section, which is equivalent to exciting carriers at the NW surface. A 2D grid of positions with 5 nm spacing are generated to represent sites which carriers can occupy. After each incremental time step, two random numbers are generated to determine the behaviour of each carrier. The first number is checked against the carrier recombination probability to establish if the carrier recombines. If recombination occurs, the carrier is removed and the recombination position is recorded. The second number determines the nearest neighbour site where the carrier moves. This process continues until each carrier recombines. This produces a distribution of positions in the cross section where the carriers have recombined, an example is shown in Figure S2a. The process is repeated for illumination from the bottom by randomly placing carriers along the bottom interface, providing a carrier distribution as shown in Figure S2b.

The Monte-Carlo simulations are repeated for a range of NW widths, carrier diffusion

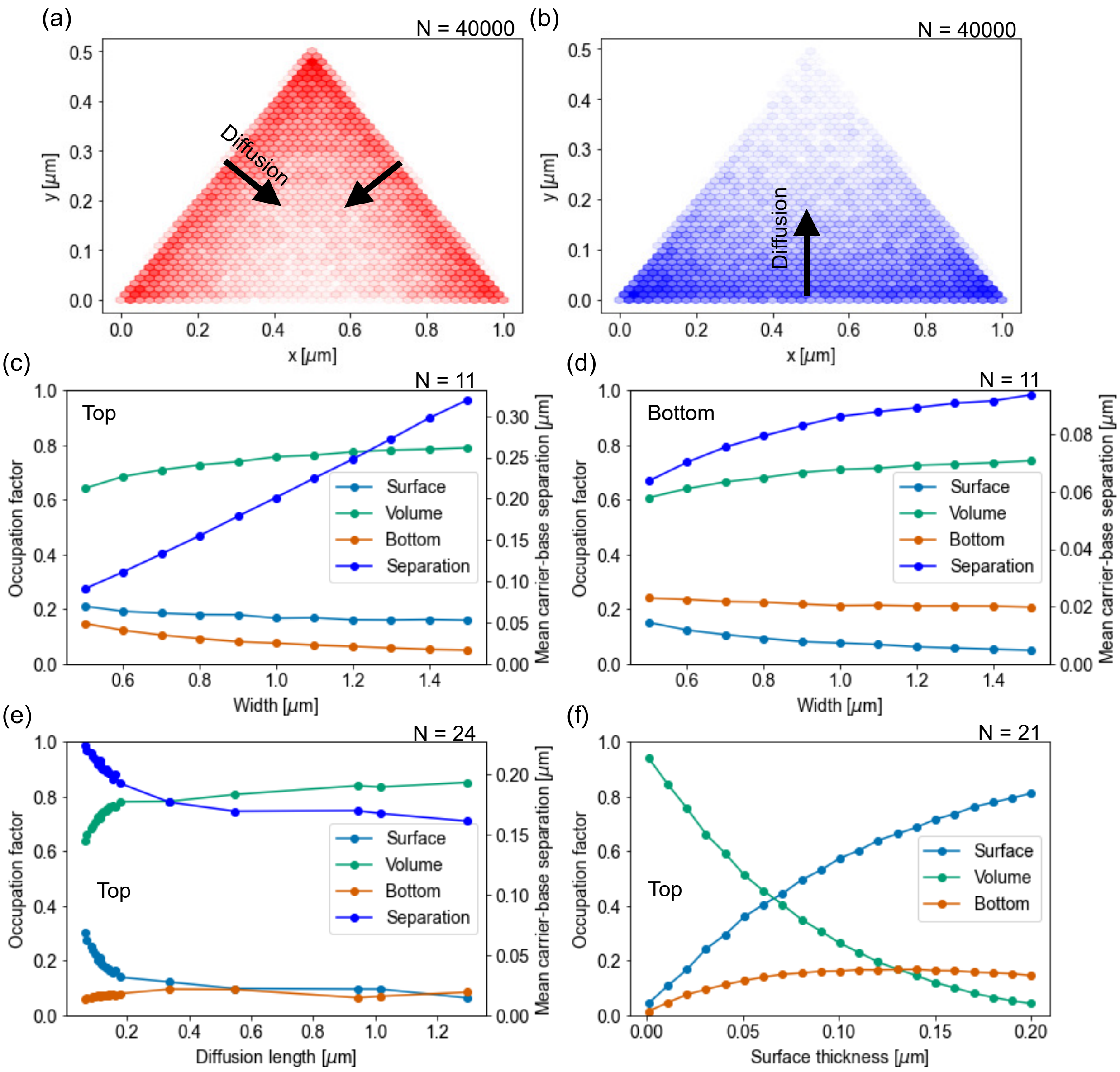

Figure S2: Summary of the results of the Monte-Carlo carrier diffusion model. (a) The distribution of carrier recombination positions when illuminating from the top of a NW with a width of 1 μm and a diffusion length of 0.15 μm. (b) The distribution of carrier recombination positions when illuminating the bottom of the same NW. (c) The variation of the carrier occupation factors at the surface, in the bulk volume and at the bottom interface when illuminating the top of NWs with different widths. The mean separation between the carrier recombination positions and the base is also shown. This calculation is for NWs with a diffusion length of 0.15 μm and a surface thickness of 10 nm. (d) The carrier occupation factors and mean separation when illuminating the bottom of the same NWs of different widths. (e) The impact of changing the diffusion length when illuminating the top of a NW with a width of 1 μm and a surface thickness of 10 nm. (f) The impact of changing the diffusion length when illuminating the top of a NW with a width of 1 μm and a diffusion length of 0.15 μm.

lengths and surface thicknesses. In each case, the proportion of carriers that are within the surface thickness from the top surface and bottom interface are calculated. The remaining carriers are in the bulk volume of the NW. These occupation factors are used, along with the geometrical volumes, to calculate the carrier densities in each of these regions. Additionally, the mean separation between the carriers and the base of the NW is calculated, which influences the effective recombination bandgap due to strain effects.

Figure S2c,d shows that, for a fixed surface thickness and diffusion length, when illuminating from the top the majority of carriers recombine in the NW bulk volume ($\approx 70\,\%$), with a reduced amount at the top surface ($\approx 20\,\%$) and even fewer from the bottom interface ($\approx 10\,\%$). This provides further justification to the assertion that the PL time decays show recombination mainly from the bulk volume, and that top surface recombination is only observed when exciting from the top of the NW. For narrow NWs the proportion of carriers in the bulk reduces due to a reducing NW volume. Reducing the NW width also reduces the carrier-base separation in a linear fashion.

When illuminating from the bottom, the majority of carriers again recombine in the bulk volume ($\approx 70\,\%$), but more carriers recombine at the bottom interface ($\approx 20\,\%$) than the top surface ($\approx 10\,\%$). In these experiments there is a large contribution from carrier recombination in the bulk volume, and a reduced contribution from recombination at the top surface.

Figure S2e shows that, for diffusion lengths longer than the NW height ($0.5\,\mu$m in this case), the carrier distribution in the NW is uniform, and so the output parameters of the model are unaffected by increasing the diffusion length. If the diffusion length is less than the NW height, carriers can no longer diffuse throughout the NW and the distribution is non-uniform, as illustrated in Figure S2a. This causes a higher proportion of carriers to be trapped at the surface, close to where they are generated, reducing the number of carriers in the bulk volume and at the bottom interface, and increasing the average carrier-base separation.

Figure S2f shows that changing the effective surface thickness causes a trivial change in the occupation factors: a thicker surface increases the proportion of carriers at the top surface and bottom interface, and reduces the number of carriers in the bulk.

The carrier diffusion length and surface thickness are fitting parameters in the carrier recombination model, which aims to fit data from >8,000 NWs with different widths. Due to limits in computational power, it is not feasible to include the full Monte-Carlo diffusion calculation in the recombination model. To circumvent this, a machine learning (ML) model is trained on the results from 13860 simulations of NWs with different widths, diffusion lengths and surface thicknesses. This ML model is capable of predicting the occupation factors and carrier-base separations with an error of <3 %. Crucially, the ML model takes $\approx 90\,\mu s$ to make these predictions, and can therefore be incorporated into a nonlinear fitting routine.

Photoluminescence Model

The room temperature photoluminescence spectra were measured using a home-built optical microscope, a schematic is shown in Figure S3a. These spectra were fit with a model which assumes that photogenerated carriers rapidly cool to the conduction and valence band edges and occupy a thermal distribution. This is reasonable for $CsPbBr_3$, as the cooling times are significantly faster than the recombination times.[6] Exciton effects can also be safely ignored at these temperatures due to a small exciton binding energy.[7] If it is assumed that the thermal energy is large enough at room temperature such that the Fermi-Dirac carrier distribution can be approximated by a Maxwell-Boltzmann distribution, the PL spectrum can be estimated by Equation S2:

$$I_{PL}(E) = G(E,\sigma) \otimes B(E)\text{exp}\left(-\frac{E-E_0}{k_\beta T}\right) \tag{S2}$$

where E_0 is the average bandgap energy and $B(E)$ is the joint density of states of the carriers.

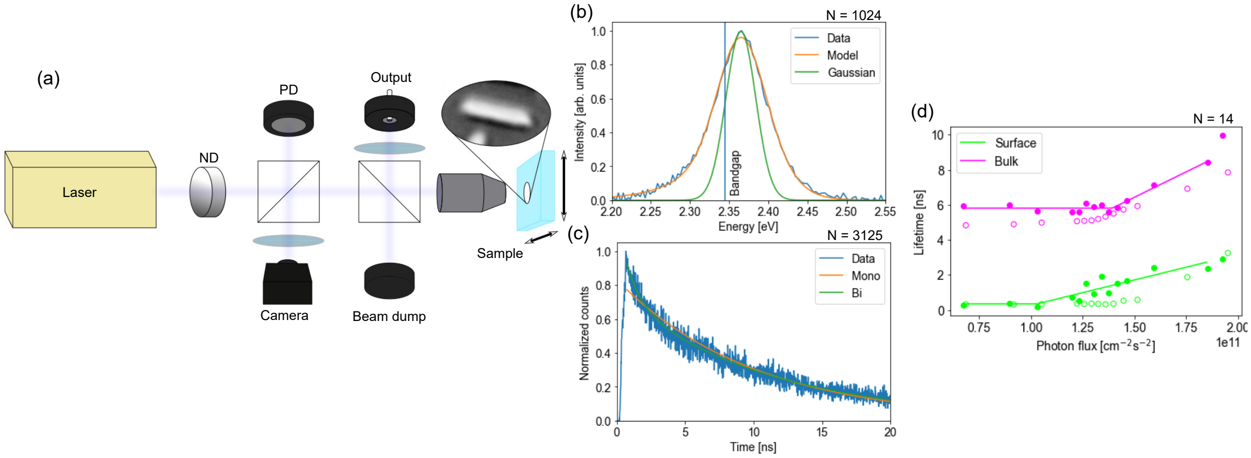

Figure S3: Optical spectroscopy and modelling of individual nanowires. (a) A schematic of the home-made microscope. Laser excitation passes through a neutral density filter (ND) and light reflects off a 50:50 beamsplitter onto a photodiode (PD) used to monitor the beam power. Transmitted light is sent to an objective lens and sample stage. Light from the sample is collected by the objective lens, is reflected by a beamsplitter, before being focused into an optical fibre. Light from the sample is also focused onto a machine vision camera for imaging. (b) Room temperature PL spectrum fit by Equation S2. The optical bandgap is indicated, which lies between the minimum and peak emission energies due to inhomogeneity in the NW. The Gaussian broadening term is also shown, which is the largest contributor to the FWHM of the emission. (c) A room temperature PL time decay measurement, showing a mono-exponential and bi-exponential fit to the data using Equation S4. The squared sum of the residuals are 13.7 and 12.8 for mono and bi-exponential decays respectively. in this case the bi-exponential fit is the most appropriate. (d) The power dependence of the PL lifetimes extracted from PL time decay fits. The full circles show the results from a typical NW and the open circles are taken from the NW with the largest fractional change in lifetime. The full circles have been fit with Equation S5 for the surface and bulk decays.

In Equation S2, G is a Gaussian function with standard deviation σ. This approximates the inhomogeneity in the NW via a convolution. A large source of this inhomogeneity is the strain, which varies with distance from the surface.[2] The strain modifies the bandgap and so carriers which recombine closer to the NW/substrate interface will have a smaller PL peak energy.

As these NWs have widths much greater than the Bohr radius,[8] the density of states is 3-dimensional. A modification to this is required at emission energies below the bandgap due to an exponential tail on the PL spectra. This is due to disorder on the atomic level in the NWs giving rise to a small density of states below the bandgap known as an Urbach tail, and is defined by an Urbach energy, E_U.[9] As a result, the density of states is given by Equation S3:

$$B(E) = \begin{cases} A_1\sqrt{E-(E_0-dE)}, & \text{if } E > E_0 - dE. \\ A_2\text{exp}\left(\frac{E-E_0}{E_\mathrm{U}}\right), & \text{if } E < E_0 - dE. \end{cases} \tag{S3}$$

where A_1 and A_2 are the amplitude of each component and dE is a small offset energy required to connect the two energy regimes.

An example of a fit to a room temperature PL spectrum from a NW is shown in Figure S3b. The average bandgap is ≈15 meV below the peak emission energy, and is ≈100 meV above the minimum emission energy. This is due to the large degree of inhomogeneity in these samples. This inhomogeneity is the major cause of the width of the PL peak, as illustrated by the Gaussian.

The Urbach tail is illustrated at the lowest energies in Figure S3b. The Urbach energy is independent of wire width: it has a Gaussian distribution centred on (12 ± 2) meV, and is comparable to the Urbach energy of $CsPbBr_3$ single crystals, where an upper limit of 19 meV was previously determined by absorption measurements.[10]

TCSPC Model

The room temperature PL time decays for each NW were measured using the same microscope system. These decays were either mono-exponential or bi-exponential in shape. All decays were fit with both a mono-exponential and a bi-exponential fit, given by Equation S4:

$$I(t) = \begin{cases} A_1 \text{exp}\left(\frac{-t}{\tau_1}\right) \\ A_1 \text{exp}\left(\frac{-t}{\tau_1}\right) + A_2 \text{exp}\left(\frac{t}{\tau_2}\right) \end{cases} \tag{S4}$$

where A is an amplitude factor and τ is the recombination lifetime for this mechanism. An example of this fitting is shown in Figure S3c. To determine the overall shape of the decay, the reduced chi-squared for each fit was compared, and the fit with the smallest chi squared was selected. The integral under the fit was also calculated - which can be used to infer the number of carriers recombining at the surface and in the bulk.

The laser excitation power was attenuated using an ND filter to measure the power dependence of the lifetimes. This experiment was performed on 220 NWs when exciting from the top. The same fitting procedure was applied to PL time decays at different excitation powers, to determine the carrier density dependence of the PL lifetimes. As shown in Figure S3d, the recombination lifetimes increase above a threshold, P_{Thresh}, which differs at the surface and the bulk. To account for this, a linear increase in the lifetimes was fit to the data using Equation S5:

$$\tau(P) = \begin{cases} C, & \text{if } P < P_{\text{Thresh}}. \\ C + m(P - P_{\text{Thresh}}), & \text{if } P > P_{\text{Thresh}} \end{cases} \tag{S5}$$

where m is the slope of the variation, and C is a lifetime offset. This linear increase is suitable because, as demonstrated in Figure S3d, we do not reach the photon flux required to saturate all of the carrier traps.

High throughput results

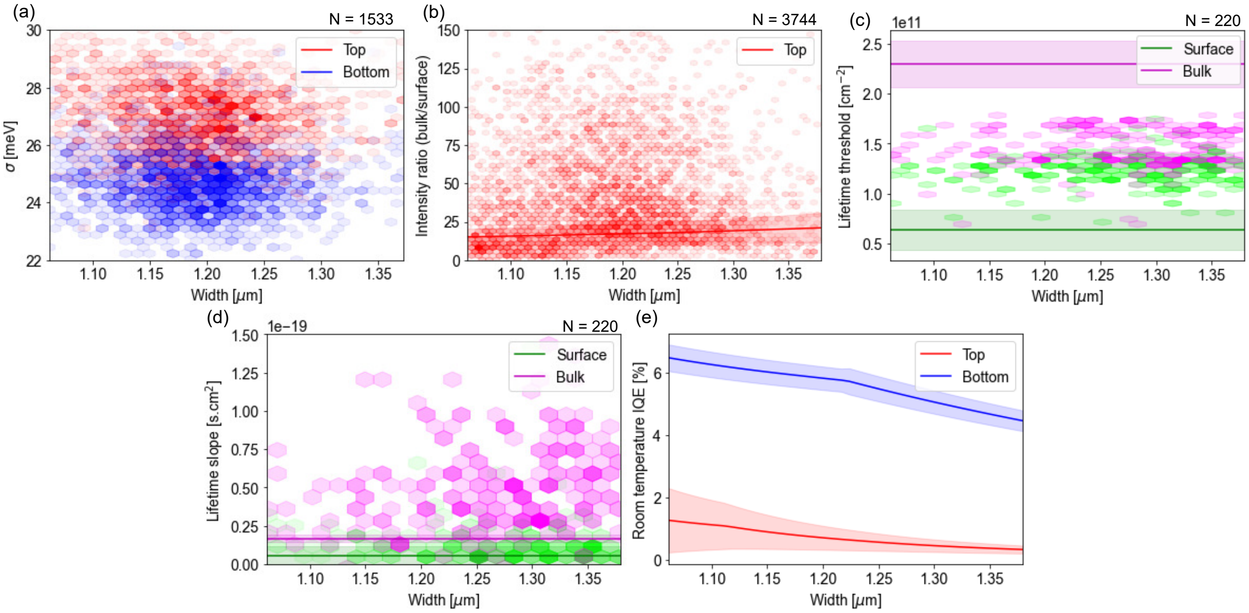

Figure S4: High throughput spectroscopy results of the NW population. (a) The variation of the PL inhomogeneity parameter σ for NWs of different widths, when excited from the top and bottom. (b) A histogram of the ratio of the integral of the TCSPC decays as determined by Equation S6 for top illumination. (c) The flux threshold above which the PL lifetime increases for both bulk and surface recombination, taken from fits to data from 220 NWs such as those in Figure S3d. (d) The slope of the lifetime variation with increasing photon flux above threshold, as described by Equation S10. (e) The variation of the recombination IQE for NWs of different widths, when excited from the top and bottom, as predicted by the model.

This section covers other results from the recombination model linking the PL, TCSPC and power dependent measurements. All fits shown are generated from the same data-constrained model as presented in the main paper and the quoted uncertainties are SEs.

The fits to the PL spectra provide the parameter σ, taken from the Gaussian convolution, which is a measure of the degree of inhomogeneity in the wire cross section. σ was determined for 1533 NWs when illuminating from the top and bottom. The variation of σ with NW width is shown in Figure S4a. These results show that σ is consistently larger for top illumination, with mean values of 27 meV and 25 meV for top and bottom illumination. This increased homogeneity when from the top is further evidence for the bandgap gradient illustrated in

Figure 1d,e: due to the triangular cross section, carriers generated near the air/NW interface are more likely to recombine in areas of different bandgap, increasing the value of σ when compared to carriers generated near the NW/substrate interface.

There is also a slight negative correlation between σ and NW width. The Pearson's correlation coefficients (r,p) are -0.1, 5E-5 and -0.08, 0.002 for top and bottom illumination respectively. Therefore, the degree of inhomogeneity increases for thinner NWs. This reflects a larger bandgap gradient across the NW cross section, which is a consequence of increased lattice rotation effects in the thinner NWs, as determined from the PL measurements in Figure 2a.

The fits to the TCSPC measurements can also be used to constrain the carrier populations in the surface and the bulk. The integral of the separate mono-exponential components in the PL time decays, I_{V} and I_{S}, are related to the carrier populations by Equation S6:

$$\frac{I_{\mathrm{V}}}{I_{\mathrm{S}}} = \frac{N_{\mathrm{V}}}{N_{\mathrm{S}}} = \frac{B_{\mathrm{V}}}{B_{\mathrm{S}}} \tag{S6}$$

where $B_{\mathrm{V,S}}$ are the occupation factors for the bulk volume and surface. These values depend on the NW width, carrier diffusion length and effective surface thickness, and are calculated using the Monte-Carlo diffusion model. This fit is shown in Figure S4b. There is no trend with optical width, due to the large degree of scatter in the data.

The power dependence of the PL lifetimes can also be used to constrain the model. The measurements shown in Figure S3d were repeated for 220 NWs: Figure S4c,d shows that both the threshold power density and lifetime slope are higher for bulk recombination, when compared with the surface, and that neither of these vary with NW width.

These results can be explained by considering the density of unoccupied traps ($n_{\mathrm{t,eff}}$) and free carriers, which are related to the trap density and the photon flux. At high photon fluxes, the trap states begin to fill up, reducing $n_{\mathrm{t,eff}}$: this is shown as an increase in the lifetime in Figure S3d. Both the threshold flux and the rate of change of the lifetime will be

dependent upon the trap density at the surface and in the bulk. Equation S7,8 assume that the threshold carrier density is proportional to the trap density:

$$n_{\mathrm{V,threshold}} = \alpha_{\mathrm{V}} n_{\mathrm{tV}} \tag{S7}$$

$$n_{\mathrm{S,threshold}} = \alpha_{\mathrm{S}} n_{\mathrm{tS}} \tag{S8}$$

where α is a proportionality factor. The threshold can be related to the threshold flux using Equation 2 in the main paper. The effective density of traps is then reduced by the density of carriers above this threshold. Equation S9 describes this for the bulk volume and top surface:

$$n_{\mathrm{t,eff}} = \begin{cases} n_{\mathrm{t}}, & \text{if } n < \alpha n_{\mathrm{t}}. \\ (1+\alpha)n_{\mathrm{t}} - n, & \text{if } n > \alpha n_{\mathrm{t}}. \end{cases} \tag{S9}$$

The rate of change of the lifetime can be calculated by differentiating Equation 5,6 with respect to the photon flux, resulting in Equation S10:

$$\frac{dt}{dn_{\mathrm{flux}}} = \frac{\alpha k_{\mathrm{t}} n_{\mathrm{t}} \langle abs \rangle \; B \langle w_{\mathrm{spot}} \rangle \; t^2}{V} \tag{S10}$$

The fits to the lifetime thresholds (Figure S4c) and lifetime slopes (Figure S4d) are the weakest part of the model. This is because these datasets have an order of magnitude fewer objects than the others, and therefore have a reduced weight in the fitting routine. The surface slope fit is within 1 σ of the data. The bulk slope fit fails to account for the large spread in the data, resulting in a fit that is 1.1 σ away from the data. The threshold fits provide the least accurate values that lie between 2.2 and 3.3 σ from the data. Nevertheless, these fits are a useful way to further constrain the global model.

The model can calculate the recombination IQE for different widths: this is shown in Figure S4e. When exciting from the top, the mean IQE extracted from the model is $(0.7 \pm 0.1)\,\%$. The IQE reduces with increasing NW width, due to a reduction in the radiative rate

and an increase in the surface non-radiative rate. As shown in Figure 3, this is due to a reduction in carrier density and a reduction in the number of saturated traps at the surface. When the NW is excited from the bottom, the mean IQE increases to $(5.8 \pm 0.4)\,\%$ due to reduced top surface recombination.

Table S1: A summary of the multi-modal data-sets produced by the high-throughput spectroscopy methodology, and the equations which describe the component of the recombination model which fits to each data-set.

Dataset	NW region	Illumination orientation	Equation
PL bandgap		Top	7
PL bandgap		Bottom	7
TCSPC lifetimes	Bulk	Top	5
TCSPC lifetimes	Surface	Top	6
TCSPC lifetimes	Bulk	Bottom	6
TCSPC lifetime threshold	Bulk	Top	S7
TCSPC lifetime threshold	Surface	Top	S8
TCSPC lifetime slope	Bulk	Top	S10
TCSPC lifetime slope	Surface	Top	S10
IQE			8
Mobility			9

As shown in Figure 2 and S4, the model can fit the full multi-modal data-set. A summary of the data-sets involved is provided in Table S1, and the output parameters are summarised in Table S2. A flowchart showing the major connections in the model is shown in Figure S5.

The diffusion length of $(0.25 \pm 0.02)\,\mu\text{m}$ is smaller than the best in class values obtained in previous studies.[11–14] This may reflect the holistic nature of this study, which involves the entire NW population, rather than focusing on the best performing. The effective surface thickness is $(6 \pm 1)\,\text{nm}$, which is roughly 10 atomic layers and is comparable to the exciton Bohr Radius in this material (7 nm[15]).

The surface trap density is $(7.1 \pm 0.3) \times 10^{16}\,\text{cm}^{-3}$, which is comparable to trap density in the bulk ($(8.6 \pm 0.4) \times 10^{16}\,\text{cm}^{-3}$). Despite this, the calculated non-radiative rate at the surface is higher than in the bulk. This is partially due to the fact that the surface traps lie within $(6 \pm 1)\,\text{nm}$ of the surface, leading to a high areal density of $(4.3 \pm 0.7) \times 10^{10}\,\text{cm}^{-2}$. The surface rate constants are also higher: The non-radiative rate is $(1.20 \pm 0.05) \times 10^{-24}\,\text{cm}^6\text{s}^{-1}$,

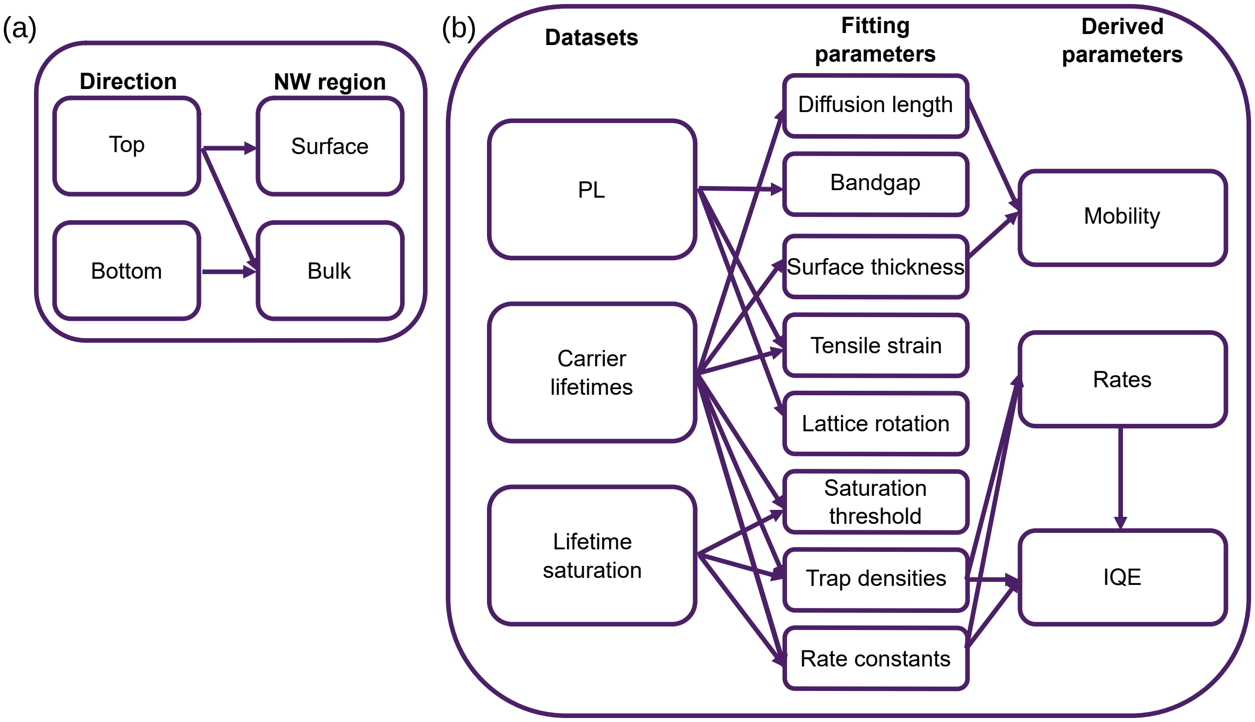

Figure S5: A flowchart summary of the holistic model. (a) The sensitivity of the measurement to different NW regions, depending upon the illumination direction. (b) The major connections between datasets, fitting parameters and derived parameters.

which is more than an order of magnitude larger than the bulk non-radiative rate of $(4.4 \pm 0.2) \times 10^{-26}\,\mathrm{cm^6 s^{-1}}$. This difference may mean that the surface traps are more likely to capture carriers. This is likely because the carriers are initially generated at the surface layer and are in close proximity to the surface traps. The rate for bulk recombination will be reduced as carriers must first diffuse to these states, and hence trapping is less likely.

The radiative recombination rate is low in comparison to the nonradiative rates, this is compatible with previous studies,[16] and is expected for these NWs where carrier traps are important.

The threshold trap density factor is a measure of the relative proportion of carriers, relative to the trap density, that are required to start to saturate the traps. The value for the bulk is (0.36 ± 0.02), and it is (0.23 ± 0.01) at the surface. This suggests that a higher proportion of carriers in the bulk are needed to saturate the traps: this result may be linked to the recombination rate of trapped carriers, suggesting that this is higher in the bulk.

Table S2: A summary of the fitting parameters used in the recombination model which was applied to the multi-modal data-set.

Parameter	Description	Fit value	unit
L_D	Diffusion length of minority carriers	(0.25 ± 0.02)	$[\mu m]$
$L_{surface}$	Effective thickness of surface layer	(6 ± 1)	[nm]
k_{rV}	Radiative recombination rate in the bulk	$(7.5 \pm 0.3) \times 10^{-27}$	$[cm^6 s^{-1}]$
k_{tV}	Nonradiative rate in the bulk	$(4.4 \pm 0.2) \times 10^{-26}$	$[cm^6 s^{-1}]$
k_{tS}	Nonradiative rate at the surface	$(1.20 \pm 0.05) \times 10^{-24}$	$[cm^6 s^{-1}]$
N_{tV}	Trap density in the bulk	$(8.6 \pm 0.4) \times 10^{16}$	$[cm^{-3}]$
N_{tS}	Trap density at the surface	$(7.1 \pm 0.3) \times 10^{16}$	$[cm^{-3}]$
α_V	Threshold trap density factor in the bulk	(0.36 ± 0.02)	
α_S	Threshold trap density factor at the surface	(0.23 ± 0.01)	
E_g	Unstrained bandgap of the NW	(2.37 ± 0.10)	[eV]
T	Bandgap shift due to tensile strain	(64 ± 3)	[meV]
L_{rot}	Variation of bandgap due to lattice rotation	(0.26 ± 0.01)	$[\mu m]$
A	Amplitude of bandgap due to lattice rotation	(1.0 ± 0.1)	[meV]

The unstrained bandgap was extracted to be (2.4 ± 0.1) eV, consistent with the literature,[17] albeit with a large uncertainty. The tensile strain at the base was determined to be (64 ± 3) meV. Literature values suggest that the bandgap shift with strain is 22 meV $\%^{-1}$,[18] therefore this model estimates that the interfacial strain is (2.9 ± 0.2) %. This is large in comparison with the strain calculated from bulk lattice parameters (0.7 %[1]). However, the thermal expansion coefficient of the $CsPbBr_3$ is an order of magnitude larger than the sapphire, which will likely lead to additional strain during post-growth cooling.[1] The NW relaxes exponentially with distance from the interface with a characteristic length of L_{rot} =(0.26 ± 0.01) µm, and amplitude of (1.0 ± 0.1) meV.

Temperature dependence of the photoluminescence

Temperature dependent PL was performed for NWs by mounting the substrate on the cold finger of a Montana cryostation. The same laser and spectrometer, as used in the main text, were also used to investigate the samples. The sample temperature was varied between 10 K and 300 K, refocusing the excitation spot at each temperature. The measurements were performed when exciting the NWs from the top and bottom, looking at 2 NWs from the top

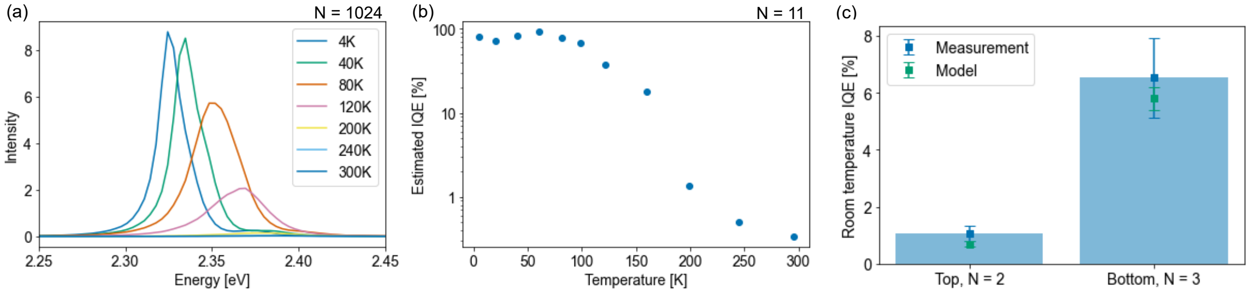

Figure S6: Temperature dependent PL results of a small number of NWs. (a) PL spectra at different temperatures for a single NW. (b) The estimated IQE of the same NW at different temperatures. (c) The weighted mean of the room temperature estimated IQE of NWs when exciting from the top and bottom.

and 3 from the bottom.

The PL spectra at different temperatures for a single NW when exciting from the top are shown in Figure S6a. At low temperature, the majority of carriers will form excitons, and recombination will be fast and highly radiative.[19] As the temperature is increased, the emission blueshifts by 42 meV due to photon-phonon coupling.[19] The excitons will also dissociate and the recombination efficiency will decrease,[12] and shallow trap states will be activated.[7] We assume that the absorption strength and hence excitation density does not vary with temperature. This is likely valid for the excitation conditions used here (405 nm) which is significantly above the band-edge and is therefore not strongly affected by excitonic absorption.[20] The ratio of the integrated intensity of the PL spectrum at low temperatures and elevated temperatures can therefore be used to estimate the IQE. This calculation was performed for the five NWs: the results for a single NW at different temperatures are shown in Figure S6b.

The room temperature estimated IQE was calculated for each NW, and a weighted mean was calculated: this is summarised in Figure S6c. When exciting from the top, the mean IQE was (1.1 ± 0.3) % - this is comparable to the predictions from the model, shown in Figure S4e. Furthermore, when exciting from the bottom of the NWs, the mean IQE increased to (7 ± 1) %.These results are both consistent with and therefore verify the capability of the model to predict optoelectronic properties of the NWs.

References

1. Oksenberg, E.; Merdasa, A.; Houben, L.; Kaplan-Ashiri, I.; Rothman, A.; Scheblykin, I. G.; Unger, E. L.; Joselevich, E. Large lattice distortions and size-dependent bandgap modulation in epitaxial halide perovskite nanowires. *Nature Communications* **2020**, *11*, 489.

2. Oksenberg, E.; Sanders, E.; Popovitz-Biro, R.; Houben, L.; Joselevich, E. Surface-Guided CsPbBr3 Perovskite Nanowires on Flat and Faceted Sapphire with Size-Dependent Photoluminescence and Fast Photoconductive Response. *Nano Letters* **2018**, *18*, 424–433.

3. Maes, J.; Balcaen, L.; Drijvers, E.; Zhao, Q.; De Roo, J.; Vantomme, A.; Vanhaecke, F.; Geiregat, P.; Hens, Z. Light Absorption Coefficient of CsPbBr 3 Perovskite Nanocrystals. *The Journal of Physical Chemistry Letters* **2018**, *9*, 3093–3097.

4. Zhao, M.; Shi, Y.; Dai, J.; Lian, J. Ellipsometric study of the complex optical constants of a CsPbBr 3 perovskite thin film. *Journal of Materials Chemistry C* **2018**, *6*, 10450–10455.

5. I. H. Malitson and M. J. Dodge., Program of the 1972 Annual Meeting of the Optical Society of America. *Journal of the Optical Society of America* **1972**, *62*, 1405.

6. Boehme, S. C.; Brinck, S. T.; Maes, J.; Yazdani, N.; Zapata, F.; Chen, K.; Wood, V.; Hodgkiss, J. M.; Hens, Z.; Geiregat, P.; Infante, I. Phonon-Mediated and Weakly Size-Dependent Electron and Hole Cooling in CsPbBr 3 Nanocrystals Revealed by Atomistic Simulations and Ultrafast Spectroscopy. *Nano Letters* **2020**, *20*, 1819–1829.

7. Baranowski, M.; Plochocka, P. Excitons in Metal-Halide Perovskites. *Advanced Energy Materials* **2020**, *10*, 1903659.

8. Berestennikov, A. S.; Li, Y.; Iorsh, I. V.; Zakhidov, A. A.; Rogach, A. L.; Makarov, S. V.

Beyond quantum confinement: excitonic nonlocality in halide perovskite nanoparticles with Mie resonances. *Nanoscale* **2019**, *11*, 6747–6754.

9. Ledinsky, M.; Schönfeldová, T.; Holovský, J.; Aydin, E.; Hájková, Z.; Landová, L.; Neyková, N.; Fejfar, A.; De Wolf, S. Temperature Dependence of the Urbach Energy in Lead Iodide Perovskites. *The Journal of Physical Chemistry Letters* **2019**, *10*, 1368–1373.

10. Rakita, Y.; Kedem, N.; Gupta, S.; Sadhanala, A.; Kalchenko, V.; Böhm, M. L.; Kulbak, M.; Friend, R. H.; Cahen, D.; Hodes, G. Low-Temperature Solution-Grown CsPbBr 3 Single Crystals and Their Characterization. *Crystal Growth & Design* **2016**, *16*, 5717–5725.

11. Stranks, S. D.; Eperon, G. E.; Grancini, G.; Menelaou, C.; Alcocer, M. J. P.; Leijtens, T.; Herz, L. M.; Petrozza, A.; Snaith, H. J. Electron-Hole Diffusion Lengths Exceeding 1 Micrometer in an Organometal Trihalide Perovskite Absorber. *Science* **2013**, *342*, 341–344.

12. Yettapu, G. R.; Talukdar, D.; Sarkar, S.; Swarnkar, A.; Nag, A.; Ghosh, P.; Mandal, P. Terahertz Conductivity within Colloidal CsPbBr 3 Perovskite Nanocrystals: Remarkably High Carrier Mobilities and Large Diffusion Lengths. *Nano Letters* **2016**, *16*, 4838–4848.

13. Lai, M.; Obliger, A.; Lu, D.; Kley, C. S.; Bischak, C. G.; Kong, Q.; Lei, T.; Dou, L.; Ginsberg, N. S.; Limmer, D. T.; Yang, P. Intrinsic anion diffusivity in lead halide perovskites is facilitated by a soft lattice. *Proceedings of the National Academy of Sciences* **2018**, *115*, 11929–11934.

14. Oksenberg, E.; Fai, C.; Scheblykin, I. G.; Joselevich, E.; Unger, E. L.; Unold, T.; Hages, C.; Merdasa, A. Deconvoluting Energy Transport Mechanisms in Metal Halide Perovskites Using CsPbBr 3 Nanowires as a Model System. *Advanced Functional Materials* **2021**, *31*, 2010704.

15. Protesescu, L.; Yakunin, S.; Bodnarchuk, M. I.; Krieg, F.; Caputo, R.; Hendon, C. H.; Yang, R. X.; Walsh, A.; Kovalenko, M. V. Nanocrystals of Cesium Lead Halide Perovskites (CsPbX 3 , X = Cl, Br, and I): Novel Optoelectronic Materials Showing Bright Emission with Wide Color Gamut. *Nano Letters* **2015**, *15*, 3692–3696.

16. Jiang, L.; Fang, Z.; Lou, H.; Lin, C.; Chen, Z.; Li, J.; He, H.; Ye, Z. Achieving long carrier lifetime and high optical gain in all-inorganic CsPbBr 3 perovskite films via top and bottom surface modification. *Physical Chemistry Chemical Physics* **2019**, *21*, 21996–22001.

17. Mannino, G.; Deretzis, I.; Smecca, E.; La Magna, A.; Alberti, A.; Ceratti, D.; Cahen, D. Temperature-Dependent Optical Band Gap in CsPbBr 3 , MAPbBr 3 , and FAPbBr 3 Single Crystals. *The Journal of Physical Chemistry Letters* **2020**, *11*, 2490–2496.

18. Li, X.; Luo, Y.; Holt, M. V.; Cai, Z.; Fenning, D. P. Residual Nanoscale Strain in Cesium Lead Bromide Perovskite Reduces Stability and Shifts Local Luminescence. *Chemistry of Materials* **2019**, *31*, 2778–2785.

19. Wolf, C.; Lee, T.-W. Exciton and lattice dynamics in low-temperature processable CsPbBr3 thin-films. *Materials Today Energy* **2018**, *7*, 199–207.

20. Diroll, B. T.; Zhou, H.; Schaller, R. D. Low-Temperature Absorption, Photoluminescence, and Lifetime of CsPbX3 (X = Cl, Br, I) Nanocrystals. *Advanced Functional Materials* **2018**, *28*, 1800945.
